# The synthesis, crystal, hydrogen sulfide detection and cell assement of novel chemsensors based on coumarin derivatives

**DOI:** 10.1038/s41598-018-34331-9

**Published:** 2018-11-01

**Authors:** Yanmei Chen, Xuefang Shang, Congshu Li, Zhenzhen Xue, Hongli Chen, Hongwei Wu, Tianyun Wang

**Affiliations:** 1 Key Laboratory of Medical Molecular Probes, School of Basic Medical Sciences, Xinxiang Medical University, Xinxiang, Henan 453003 China; 2 School of Pharmacy, Xinxiang Medical University, Jinsui Road 601, Xinxiang, Henan 453003 China; 3 School of Life Sciences and Technology, Xinxiang Medical University, Jinsui Road 601, Xinxiang, Henan 453003 China; 4 Department of biochemistry, Xinxiang Medical University, Jinsui Road 601, Xinxiang, Henan 453003 China

## Abstract

A series of chemsensors (**1**–**4**) containing fluorobenzene group based on coumarin derivatives have been developed for the selective and sensitive detection of H_2_S. The advantages of the synthesized fluorescent probe (compound **1**) were the low detection limit (4 × 10^−6^ mol·L^−1^), good selectivity and high sensitivity which had been demonstrated through UV-vis, fluorescent titration experiments. Besides cytotoxicity test of compounds (**1** and **2**) was studied and the results indicated that compounds (**1** and **2**) showed almost no cytotoxicityat at a concentration of 150 μg·mL^−1^. The interacted mechanism was the thiolysis reaction of dinitrophenyl ether which had been confirmed by fluorescence and HRMS titration experiment. In addition, probe **1** can also detect HS^−^ selectively by naked eye in pure DMSO solvent.

## Introduction

In the past decade, we has seen a boost of research interest in hydrogen sulfide (H_2_S), a colorless, flammable, toxic gas with unpleasant smell, which is recognized as a signal gasotransmitter in the body as same as nitric oxide (NO)^1–10^ and carbon monoxide (CO)^11^. Endogenous concentration of H_2_S is related to some diseases such as Alzheimer’s disease, Down syndrome, liver cirrhosis and diabetes^2,9,12–17^. What’s more, the regulation of H_2_S levels is also a potential drug development strategy^18,19^ and the importance of accurate detection of H_2_S cannot be over-emphasized. Therefore, it presents significant research related to track and quantify H_2_S inside living cells being crucial in order to understand the biological and pathological roles of H_2_S. Recently, some methods to determine H_2_S concentration in biological sample have been developed including the methylene blue, the monobromobimane (MBB), gas chromatography (GC), the sulphide ion selective electrodes (ISE) and fluorescent analysis^20–23^. Among these methods, fluorescent analysis has attracted great attention due to the high sensitivity and selectivity for the detection of H_2_S in many fields such as environment area, pharmacy area and so on^24–31^.

**Figure 1:**
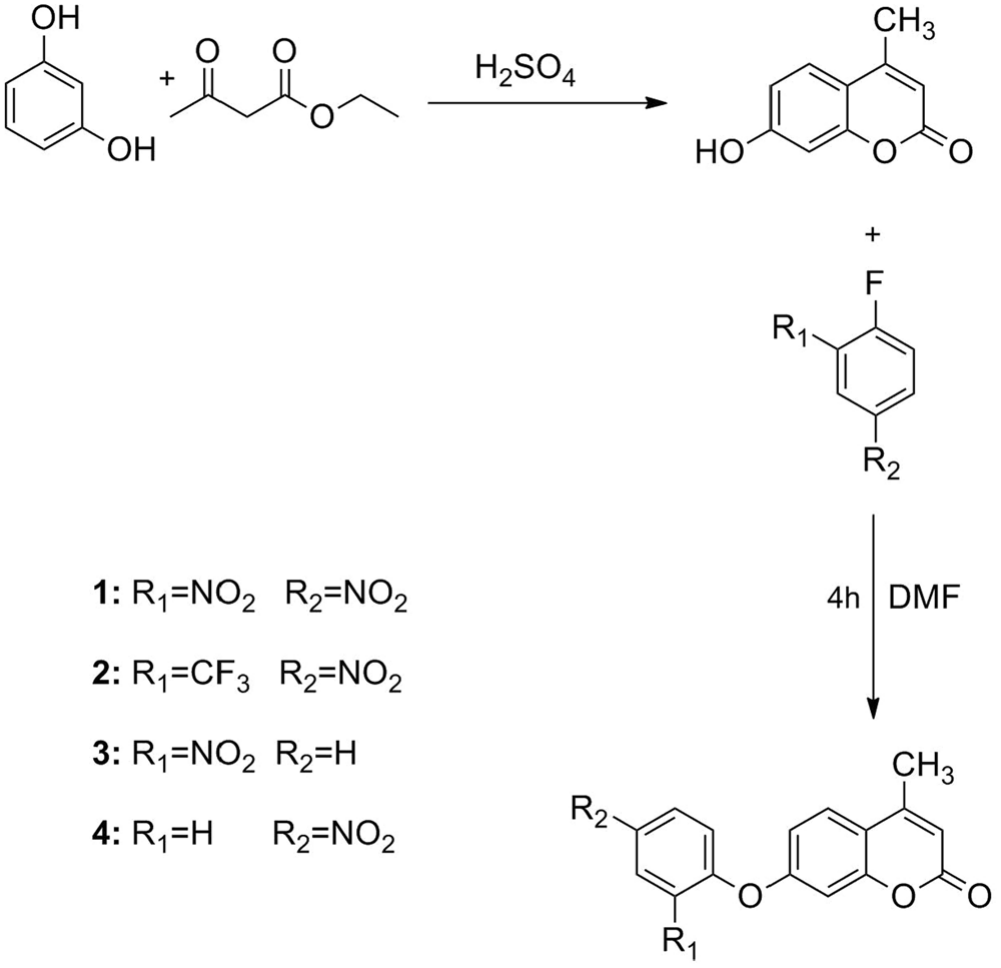
The synthesis route of the probes (**1**–**4**).

In the present work, a series of “OFF-ON” probes based on coumarin derivatives to detect HS^−^ (Fig. 1). The results of UV-vis titration experiments indicated that the synthesized compounds showed high binding ability for HS^−^ among the tested anions (NaHS (HS^−^), (*n*-C_4_H_9_)_4_NAcO (AcO^−^), (*n*-C_4_H_9_)_4_NH_2_PO_4_ (H_2_PO_4_^−^), (*n*-C_4_H_9_)_4_NF (F^−^), (*n*-C_4_H_9_)_4_NCl (Cl^−^), (*n*-C_4_H_9_)_4_NBr (Br^−^), (*n*-C_4_H_9_)_4_NI (I^−^)) and amino acids (Glutathione (GSH), Cysteine (Cys), Homocysteine (Hcy)). Besides, four compounds designed and synthesized could exhibit the probes with strong electron-withdrawing groups located in 2,4-positions of fluorobenzene have strong binding ability for HS^−^ detection which provides a good idea for the design of probe in future.

## Results and Discussion

### X-ray crystallography

Compound **2** was synthesized according to the route shown in Fig. 1. Fortunately, the crystallographic of compound **2** was obtained by the standing method. The suitable single light yellow crystal was obtained by volatilizing ethyl acetate containing compound **2** at room temperature. The details of the crystallographic determination, selected bond lengths and angles were given in Table 1 and supplementary material respectively.

**Table 1:** Crystal data and structure refinement for Compound **2**.

Compound 2	
Empirical formula	C_17_ H_10_ F_3_ N O_5_
Formula Weight	365.26
Temperature	293 K
Bond precision	C-C = 0.0031 A
Wavelength	0.71073
*a* (Å)	7.7988(10)
*b* (Å)	8.2075(11)
*c* (Å)	13.5474(14)
*α* (°)	75.073(10)
*β* (°)	81.40(1)
*γ* (°)	69.207(12)
*V* (Å^3^)	781.69(17)
Crystal system, Space group	Monoclinic, P-1
Crystal size (mm^3^)	0.45 × 0.32 × 0.23
θ range for data collection (°)	3.19 to 25.01
Z	2
Mu (mm^−1^)	0.138
*F*(000)	372.0
Refinement method	Full-matrix least-squares on F^2^
GOF on F^2^	1.027
*R*_1_ and w*R*_2_ indices [*I* > 2σ(*I*)]	*R*_1_ = 0.0446, w*R*_2_ = 0.1144
*R*_1_ and w*R*_2_ indices (all data)	*R*_1_ = 0.0645, w*R*_2_ = 0.1304
Largest diff. peak and hole (e Å^−3^)	0.258 and −0.250

**Figure 2:**
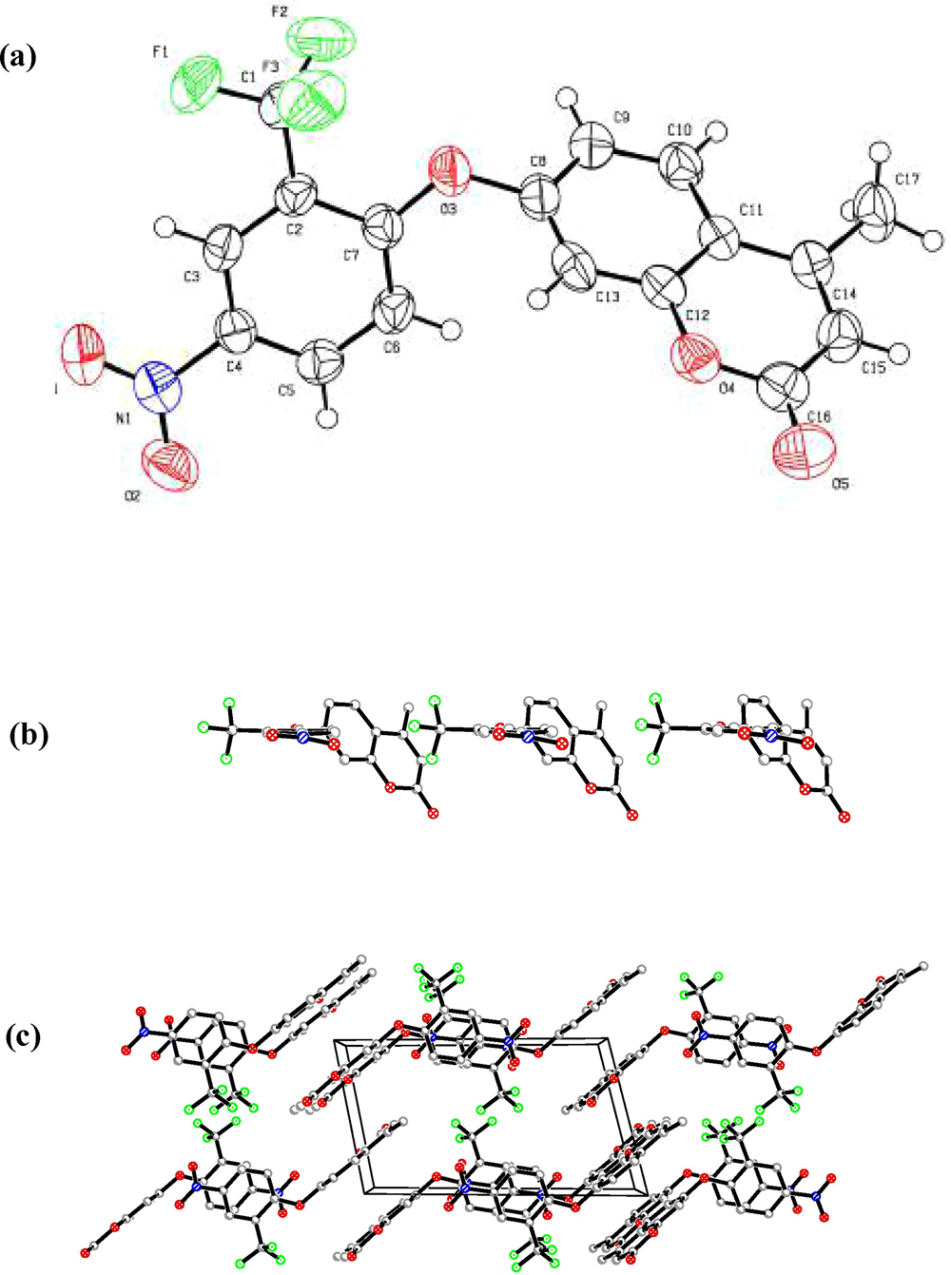
(**a**) The ORTEP view of compound **2** and the hydrogen atoms are shown as small circles with arbitrary radii (ellipsoids at 50% probability); (**b**) Crystal packing of compound **2** along the **b** axis; (**c**) Crystal packing of compound **2** along the *a* axis.

The crystal of compound **2** suitable for X-ray crystal analysis was obtained and the structure was also confirmed (Fig. 2a). The fluoride atom in benzene cycle forms hydrogen bonds with hydrogen atom (H3) (supplementary material). The overall crystal structure features a chain type joining in through the hydrogen bonds (H….F) along the *b* axis (Fig. 2b**)**. In the crystal packing of compound **2** (Fig. 2c), there are two stacked forms: (1) π-π stacking of one fluorobenzene ring with another; (2) π-π stacking of one coumarin ring with another, which connected into “chair” conformation along *a* axis.

### UV-vis Titration

**Figure 3:**
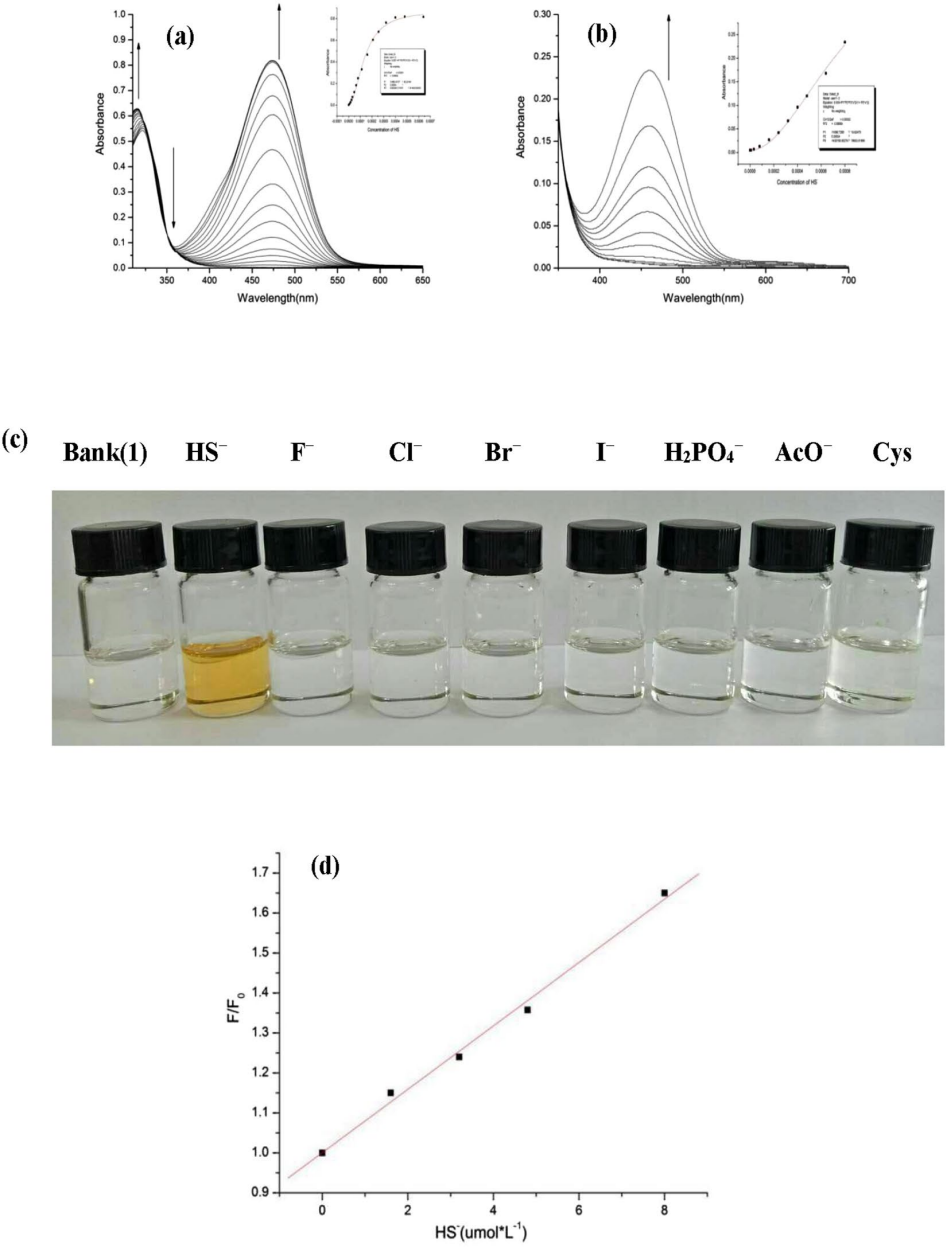
UV-vis spectra of compound **1** (4.0 × 10^−5^ mol·L^−1^) with the addition of HS^−^(0–8 × 10^−6^ mol·L^−1^)(**a**) in DMSO solution; (**b**) in aqueous solution (DMSO-H_2_O 4:1, v/v 0.04 mol·L^−1^ HEPES buffer at pH 7.38). Arrows indicate the direction of increasing HS^−^ concentration; (**c**) Color changes observed with the addition of 20 equiv. various anions and Cys to DMSO solution of compound **1** respectively; (**d**) Fluorescent intensity of compound **1** upon the addition of HS^−^.

The UV-vis spectra of probes (**1**–**4**) were recorded after addition of amino acids (GSH, Cys, Hcy) and various anions (HS^−^, AcO^−^, H_2_PO_4_^−^, F^−^, Cl^−^ Br^−^ and I^−^) through UV-vis titration experiments in pure DMSO solution and aqueous solution (DMSO-H_2_O 4:1, v/v 0.04 mol·L^−1^ HEPES buffer at pH 7.38) respectively. The data of UV-vis titration experiments manifested only compounds (**1**, **2**) displayed different binding abilities with the above anions and amino acids. The free **1** showed a main absorption at 320 nm, as the HS^−^ increases in pure DMSO solution of probe **1**, the absorbance at 320 nm was decreased gradually, along with the simultaneous emergence of a new absorption at 470 nm. In this process, two isosbestic points noted at 330 nm and 348 nm suggesting a clear chemical reaction. Based on the well-establish thiolysis reaction of dinitrophenyl ether, the new absorption at 470 nm could be attributed to coumarin derivative, which was also supported by fluorescence and HRMS titration experiment. Furthermore, the UV-vis spectra of probe **1** with HS^−^ in aqueous solution were also performed (shown in Fig. 3b), however, comparison with DMSO solvent, the probe **1** showed a weak response of UV-vis spectra.

The additions of amino acids (Cys, GSH, Hcy) and other anions (AcO^−^, H_2_PO_4_^−^, F^−^, Br^−^, Cl^−^ and I^−^) to pure DMSO solution of probe **1**, only Cys induced similar changes in the UV-vis spectra compared with HS^−^, which exhibited Cys also interacted with compound **1** (supplementary material). However, the additions of the above amino acids and anions to aqueous solution induced almost no spectra changes of compound **1**. The result indicated that compound **1** showed different binding abilities for HS^−^ and Cys in pure DMSO solution and almost no binding abilities with above amino acids and anions in aqueous solution. Therefore, compound **1** could be used as a sensor to detect HS^−^ in aqueous solution.

Subsequently, the colorimetric sensing capabilities of compound **1** were carried out with different anions (Fig. 3c). Obvious color changes from colorless to bright yellow was observed in the presence of HS^−^, while faint or no color changes happened in the presence of other anions (AcO^−^, H_2_PO_4_^−^, F^−^, Cl^−^, Br^−^, I^−^ and Cys), which indicated that compound **1** can be used for the detection of HS^−^ as a colorimetric sensor.

Moreover, the detection limit^32,33^ of compound **1** (4.0 × 10^−6^ mol·L^−1^) was implemented through fluorescence titration (Fig. 3d). Further data analysis revealed an excellent linear relationship (r = 0.9963) between the fluorescence signal of the probe **1** at 392 nm and the concentration of HS^−^ (0–8 × 10^−6^ mol·L^−1^). Therefore, the detection limit of compound **1** for HS^−^ was determined to be 4.0 × 10^−6^ mol·L^−1^.

Next, the UV-vis titration experiments of compound **2** to various anions (HS^−^, AcO^−^, H_2_PO_4_^−^, F^−^, Br^−^, Cl^−^ and I^−^) were tested. Upon the addition of increasing amounts of HS^−^ (supplementary material) to DMSO solution of compound **2** a new absorption peak appeared gradually at 485 nm. While, the additions of other anions (AcO^−^, H_2_PO_4_^−^, F^−^, Br^−^, Cl^−^ and I^−^) to compound **2**, almost no spectra changes was observed.

### Fluorescence response

**Figure 4:**
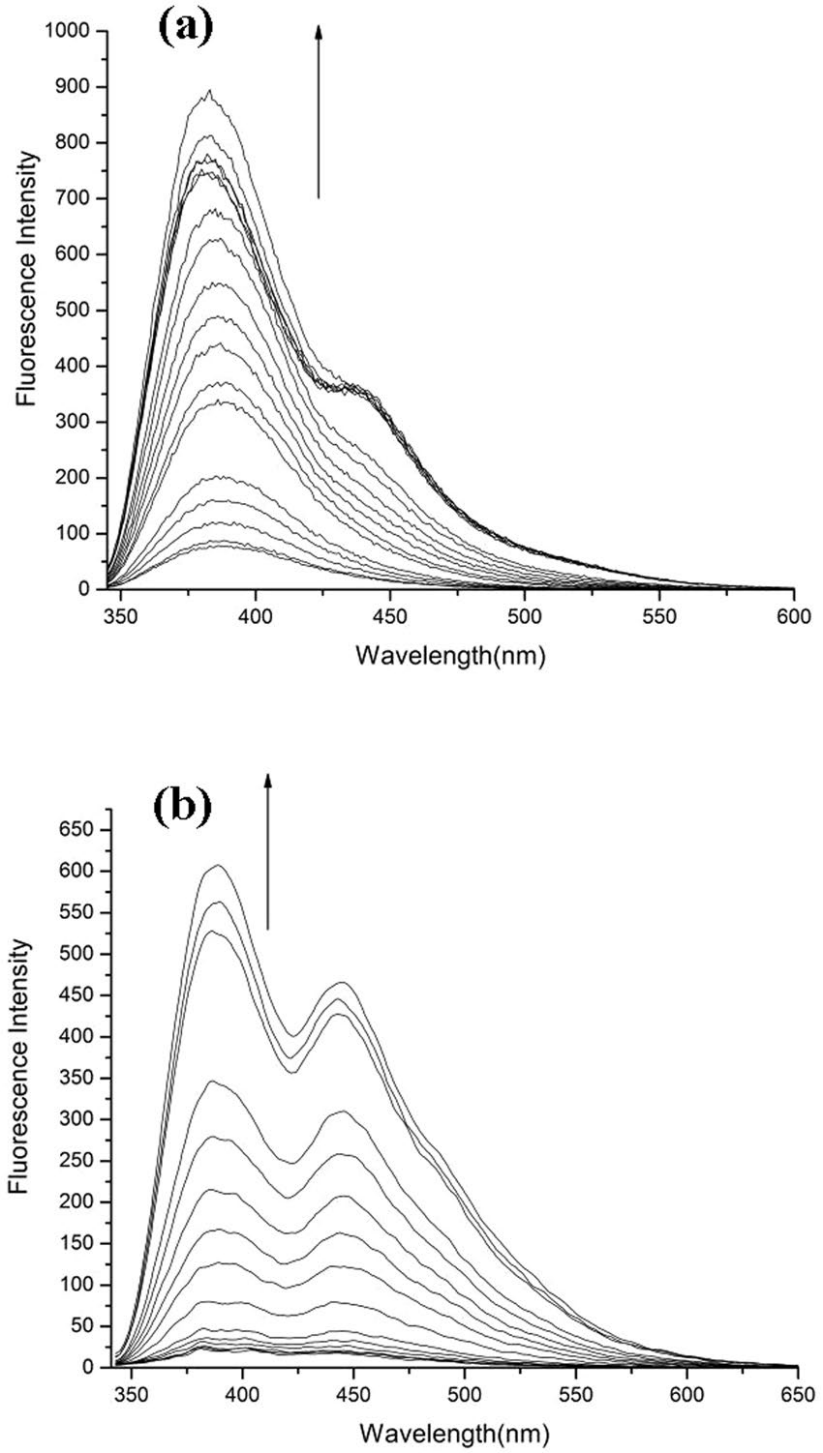
Fluorescence responses (λ_ex_ 331 nm, slit widths: 5 nm/5 nm) of compound **1** (4.0 × 10^−5^ mol.L^−1^) upon the additions of HS^−^ (**a**) in pure DMSO solution; (**b**) in aqueous solution (DMSO-H_2_O 4:1, v/v 0.04 mol·L^−1^ HEPES buffer at pH 7.38). Arrows indicate the increasing concentration of HS^−^.

The photo-physical responses of four compounds (**1**–**4**) in DMSO solvent were also investigated with addition of various amino acids and anions. Just as Fig. 4a showed, an emission peak of free **1** exhibited at about 385 nm. Upon the addition of increasing amounts of HS^−^, the fluorescence intensity increased obviously at about 392 nm. Similar fluorescence response of compound **1** was observed upon the addition of Cys (supplementary material) compared with HS^−^. Furthermore, the interactions of probe **1** with amino acids (GSH, Hcy) and other anions (AcO^−^, H_2_PO_4_^−^, F^−^, Br^−^, Cl^−^ and I^−^) were also investigated. The additions of H_2_PO_4_^−^, AcO^−^ and F^−^ (supplementary material) induced the appearance of fluorescence emission bands centered at about 386 nm, however, nominal changes were induced in the presence of GSH, Hcy, F^−^, Cl^−^, Br^−^, I^−^.

Besides, the fluorescence spectral responses of compound **1** with amino acids (Cys, GSH, Hcy) and various anions (HS^−^, AcO^−^, H_2_PO_4_^−^, F^−^, Br^−^, Cl^−^ and I^−^) were also examined in aqueous solution. From Fig. 4b, compound **1** showed very weak fluorescence at the absence of anions. After HS^−^ was added, double emission peaks appeared and the fluorescent intensity increased gradually. Results demonstrated that compound **1** showed strong binding ability for HS^−^. However, weak spectral responses that even could be ignored were induced with addition of amino acids (Cys, GSH, Hcy) and H_2_PO_4_^−^, AcO^−^, F^−^, Cl^−^, Br^−^, I^−^.

For compound **2**, the fluorescence intensity noted at 434 nm increasing rapidly by titration of HS^−^ (supplementary material). No obvious responses of compound **2** were observed with titration of other anions (AcO^−^, H_2_PO_4_^−^, F^−^, Br^−^, Cl^−^ and I^−^**)** and Cys. For compounds (**3** and **4**), Similar experiments were carried out, however, no significant spectral responses were observed with the addition of other anions (HS^−^, H_2_PO_4_^−^, AcO^−^, F^−^, Br^−^, Cl^−^ and I^−^) into DMSO solution of two compounds (**3** and **4**) which indicated the weak binding abilities of compounds (**3** and **4**) and other anions (AcO^−^, H_2_PO_4_^−^, F^−^, Br^−^, Cl^−^ and I^−^**)** and Cys could be ignored.

### Binding constant

The job-pot curves suggested two compounds (**1** and **2**) interacted with amino acids and various anions as the ratio of 1:1 or 1:2. The UV-vis spectral data was used to calculate the binding constants by non-linear least square method^34,35^, and the binding constants were listed in the Table 2. Obviously, the binding ability of two compounds (**1** and **2**) with amino acids and various anions followed the order of HS^−^ ≫ H_2_PO_4_^−^, Cys, AcO^−^, F^−^, Br^−^,Cl^−^, I^−^, Hcy and GSH. In general, both compound **1** and compound **2** showed the strongest binding ability for HS^−^ among amino acids and anions. Besides, a theoretical basis and these binding constants were necessary for the optimization of sensor.

**Table 2:** Binding constants of compound (**1** and **2**) with various anions.

Anion	Compound 1 (DMSO)	Compound 1 (DMSO:HEPES = 4:1)	Compound 2 (DMSO)
HS^−^	(5.32 ± 0.08) × 10^7b^	(1.43 ± 0.07) × 10^6b^	(33.9 ± 0.8)^a^
Cys	(7.91 ± 0.64) × 10^3a^	ND	ND
AcO^−^	ND	ND	ND
F^−^	ND	ND	ND
H_2_PO_4_^−^	ND	ND	ND
Cl^−^ Br^−^ or I^−^	ND	ND	ND

^a^The binding ratio of host-guest is 1:1.

^b^The binding ratio of host-guest is 1:2.

The anion binding abilities of compounds (**1** and **2**) with two electron-withdrawing groups on the fluorobenzene ring were stronger than that of compounds (**3** and **4)** which had one electron-withdrawing group on the fluorobenzene ring. In addition, the anion binding ability of compound **1** was stronger than that of compound **2** due to the electron-withdrawing ability of nitro group was greater than the trifluoromethyl group^36^. The above results indicated that strong electron-withdrawing groups located in 2, 4-positions of fluorobenzene provides a easily site for intermolecular HS^−^ attack. Besides, for HS^−^, the binding ability trend of compounds (**1–4**) followed the order of **1** > **2** ≫ **3**, **4** also could be confirmed by comprehensive analysis of UV-vis, fluorescence titration.

### Mechanism

**Figure 5:**
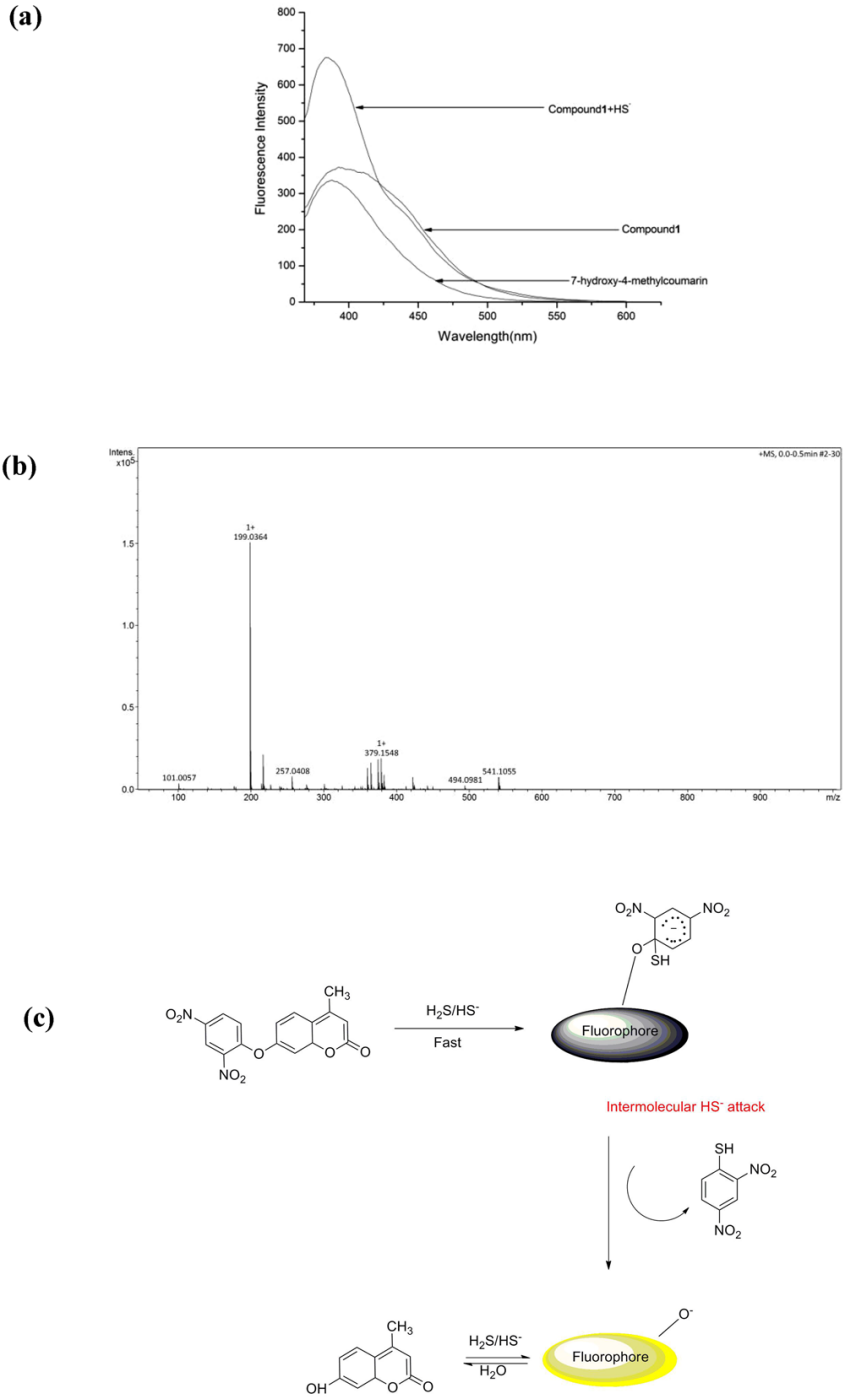
(**a**) A comparison of fluorescence spectra in the presence of 3.2 equiv HS^−^ between compound **1** and 7-hydroxy-4-methylcoumarin (4.0 × 10^−5^ mol·L^−1^); (**b**) ESI-HRMS spectrum of compound **2** after addition of 2 equiv of NaHS in DMSO solution. MS-HRMS (m/z): 199.0364 (*M* + Na)^+^; (**c**) The possible interacted mechanism of host-guest.

The interacted mechanism was measured by fluorescence and HRMS titration experiment. The broad emission peak of compound **1** appeared at about 396 nm in the absence of HS^−^. The emission peak shifted to the short wavelength from 396 nm to 380 nm after HS^−^ was added. The emission peak of the single 7-hydroxy-4-methylcoumarin appeared at about 380 nm. The same emission peak observed in the presence of HS^−^ which suggested free 7-hydroxy-4-methylcoumarin was released after compound **1** interacted with HS^−^ (Fig. 5a). Furthermore, the interacted mechanism of host-guest (**1**-HS^−^) was also carried by performing MS-HRMS after the addition of 2 equiv. NaHS. The probe **1** itself exhibited a dominant peak at m/z = 341.0551(*M*-H)^−^ (supplementary material). However, the above peak vanished and a new peak at m/z = 199.0364 (*M* + Na)^+^ appeared which was the ion-peak of 7-hydroxy-4-methylcoumarin (Fig. 5b) after the addition of HS^−^. The MS-HRMS titration experiment suggested the binding ratio of host-guest (**1**-HS^−^) was 1:2. The above results indicated the possible interacted mechanism was thiolysis reaction of dinitrophenyl ether (Fig. 5c)^37–44^.

### Cytotoxicity Assay

**Figure 6:**
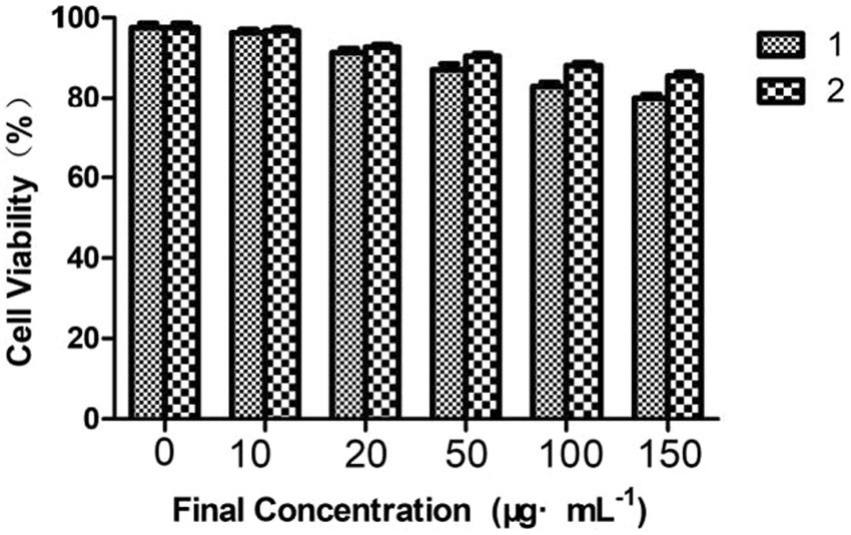
Cell viabilities were measured by MTT assay after 24 h in the presence of of fluorescence probe (0–150 μg·mL^−1^) incubation. Cell viability (expressed in%) was set as 100% growth in the absence of fluorescence probe.

For further biological application point of view, a quantitative cytotoxicity study of two compounds (**1** and **2)** *in vitro* was conducted using MTT assay. Owning to Glutathione peroxidase (GPx1) being an important selenoprotein and not found in human breast cancer cells (MCF-7) according to scientific research, therefore, the special cell lines (MCF-7 cell) were selected by us^45,46^. The MTT assay results indicated that compound **1** and **2** showed very low cototoxicity over a concentration range of 0–150 μg·mL^−1^ especial probe **2** (Fig. 6). Cellular viability was minimally affected (80%, cellular viability) with the compounds (**1** and **2**) ( <150 μg·mL^−1^). In agreement with the binding constants, compounds (**1** and **2**) showed a high binding capacity and low cytotoxicity, especial probe **1**, which indicated that compounds (**1** and **2**) have a potential application to H_2_S detection in cells.

## Material and Methods

Most staring materials were obtained commercially, all reagents and solvents were of analytical grade and used as received without further purification unless otherwise stated. Sodium hydrolfide and all anions in the form of tetrabutylammonium salts (such as (*n*-C_4_H_9_)_4_NCl, (*n*-C_4_H_9_)_4_NBr, (*n*-C_4_H_9_)_4_NI, (*n*-C_4_H_9_)_4_NAcO and (*n*-C_4_H_9_)_4_NH_2_PO_4_) and amino acides (Cys, GSH and Hcy) were purchased from Aladdin Chemistry Co. Ltd (Shanghai, China). Dimethyl sulfoxide (DMSO) was distilled in vacuum after being dried with CaH_2_. 1H NMR spectra were recorded using an Unity Plus-400-MHz spectrometer. HRMS was obtaioned with a Mariner apparatus. Absorption spectra were obtained on UV-vis spectrophotometer (Shimadzu, UV-2600, Japan). Fluorescence emmission spectra were taken on a Cary Eclipse Fluorescence Spectrophotometer (Agilent, USA). The binding constant (*K*_s_) was obtained by non-linear least squares calculation method for data fitting.

The cells that were at logarithmic growth phase were seeded in a 96-well plate at a density of 2.0 × 10^4^ cell peer well for 24 h, followed by treatment different concentration of compound at 37 °C for additional 24 h. Next, cells were collected and washed with PBS three times then 100 µL of culture medium and 20 µL of MTT solution were added to each well respectively, and the cells were incubated for 4 h. The absorbance were detected by microplate reader (Thermo Multiscan MK3, Thermo Fisher Scientific, MA, USA) at 490 nm wavelength measurement. Cell viability (expressed in%) was calculated considering 100% growth at the absence of fluorescence probe, and the viability of other groups were calculated by comparing the optical density reading with the control. The IC50 was defined as the compound concentrations required for 80% inhibition of cell growth.

### Synthesis of the probes

The compounds (**1**–**4**) were synthesized according to the route shown in Fig. 1.

#### Synthesis of 7-hydroxy-4-methylcoumarin (**1**)

7-Hydroxy-4-methylcoumarin was synthesized according to the literature^47^. The experimental details were described in supplementary material.

#### Synthesis of compound **1**

7-Hydroxy-4-methyl coumarin (2.16 g, 12 mmol) and 2,4-dinitrofluorobenzene (1.5 g, 8 mmol), K_2_CO_3_ (3.39 g, 24 mmol) were dissolved in DMF(40 mL) solution with stirring. The above mixture were heated for 4 h at 90 °C at N_2_ atmosphere and then cooled to room temperature. The reaction was poured into ice-water (400 mL) and extracted with ethyl acetate (3 × 25 mL), dried with MgSO_4_ overnight. Filtration, purification with column chromatography (CH_3_COOCH_2_CH_3_:CH_2_Cl_2_ = 1:40). Stood overnight a yellow precipitate was obtained. Yield: 68%. m.p. 187.9–189.8 °C. ^1^H NMR (400 MHz, DMSO-*d*_6_) δ 8.94 (s, 1 H), 8.51 (dd, *J* = 9.2, 2.8 Hz, 1 H), 7.92 (d, *J* = 8.7 Hz, 1 H), 7.40 (dd, *J* = 15.6, 5.8 Hz, 2 H), 7.28 (dd, *J* = 8.7, 2.5 Hz, 1 H), 6.43 (s, 1 H), 2.47 (s, 3 H) (supplementary material). Elemental analysis: Calc. for C_16_H_10_N_2_O_7_: C, 56.15; H, 2.94; N, 8.18. Found: C, 56.21; H, 2.94; N, 8.16. MS-HRMS (m/z): 341.0551(*M*-H)- (supplementary material). IR: C-O-C (Diphenyl oxide Phenyl ether Biphenyloxide): 1288 cm^−1^ (supplementary material).

Compounds (**2**–**4**) were also synthesized according to the above similar procedure.

#### Compound **2**

Suitable single light yellow crystal for X-ray crystal structure analysis was obtained. Yield: 74%. m.p. 139.3–142.1 °C. ^1^H NMR (400 MHz, DMSO-*d*_6_) δ 8.56 (d, *J* = 2.8 Hz, 1 H), 8.51 (dd, *J* = 9.1, 2.8 Hz, 1 H), 7.91 (d, *J* = 8.7 Hz, 1 H), 7.39–7.30 (m, 2 H), 7.23 (dd, *J* = 8.7, 2.5 Hz, 1 H), 6.42 (s, 1 H), 2.46 (s, 3 H) (supplementary material). Elemental analysis: Calc. for C_17_H_10_FNO_5_: C, 55.90; H, 2.76; N, 3.83. Found: C, 55.83; H, 2.76; N, 3.84. MS-HRMS (m/z): 366.0589(*M* + H)^+^ (supplementary material). IR: C-O-C (Diphenyl oxide Phenyl ether Biphenyloxide): 1274 cm^−1^ (supplementary material).

#### Compound **3**

Yield: 75%. m.p. 140.9–143.6 °C. ^1^H NMR (400 MHz, DMSO-*d*_6_) δ 8.16 (dd, *J* = 8.2, 1.5 Hz, 1 H), 7.86–7.76 (m, 2 H), 7.51 (t, *J* = 7.8 Hz, 1 H), 7.39 (d, *J* = 8.3 Hz, 1 H), 7.09–7.01 (m, 2 H), 6.35 (s, 1 H), 2.43 (s, 3 H) (supplementary material). Elemental analysis: Calc. for C_16_H_11_NO_5_: C, 64.65; H, 3.73; N, 4.71. Found: C, 64.72; H, 3.72; N, 4.72. MS-HRMS (m/z): 320.0550(*M* + Na)^+^ (supplementary material). IR: C-O-C (Diphenyl oxide Phenyl ether Biphenyloxide): 1273 cm^−1^ (supplementary material).

#### Compound **4**

Yield: 78%. m.p. 165.5–167.3°C. ^1^H NMR (400 MHz, DMSO-*d*_6_) δ 8.35–8.27 (m, 2 H), 7.88 (d, *J* = 8.7 Hz, 1 H), 7.28 (dd, *J* = 10.2, 3.1 Hz, 3 H), 7.19 (dd, *J* = 8.7, 2.4 Hz, 1 H), 6.40 (s, 1 H), 2.46 (s, 3 H) (supplementary material). Elemental analysis: Calc. for C_16_H_11_NO_5_: C, 64.65; H, 3.73; N, 4.71. Found: C, 64.82; H, 3.74; N, 4.70. MS-HRMS (m/z): 320.0533 (*M* + Na)^+^ (supplementary material). IR: C-O-C (Diphenyl oxide Phenyl ether Biphenyloxide): 1267 cm-1 (supplementary material).

A light yellow crystal of compound **2** with dimensions of 0.45 nm × 0.32 nm × 0.23 nm was mounted on a glass fiber. X-ray single-crystal diffraction data was collected on a Rigaku saturn CCD area detector at 293 K with Mo-Ka radiation (λ = 0.71073 Å). The structure was solved by direct methods and refined on *F*^2^ by full-matrix least squares methods with SHELXL-97^48^.

## Conclusion

In conclusion, we developed a series of fluorescence probes based on coumarin derivatives for the detection of H_2_S successively with OFF-ON” fluorescence response. The fluorescence probes, especially compound **1**, exhibited remarkable response to H_2_S against other anions and amino acids in pure DMSO solvent. Otherwise, the probe **1** also showed strong binding ability for HS^−^ in HEPES buffer solution. The interacted mechanism of host-guest was the thiolysis reaction of dinitrophenyl ether. In addition, compound (**1** and **2**) showed highly sensitivity and low cytotoxicity to MCF-7 cells and probe **1** can also detect HS^−^ selectively by naked eye in pure DMSO solvent. The results of our efforts highlight that the probes, especially compound **1**, hold a potential chemical tool for the detection of H_2_S.

## Electronic supplementary material

Supplementary Information
