# Supplementary material for: The synthesis, crystal, hydrogen sulfide detection and cell assement of novel chemsensors based on coumarin derivatives
Source: Sci Rep. 2018 Nov 1;8:16159. doi: 10.1038/s41598-018-34331-9 (PMC6212500; doi:10.1038/s41598-018-34331-9)
Supplement: Supplementary file 1 — Supplementary Information [file 41598_2018_34331_MOESM1_ESM.pdf]

The synthesis, crystal, hydrogen sulfide detection and cell assement of novel chemsensors based on coumarin derivatives

Yanmei Chen[a], Xuefang Shang[a]*, Congshu Li[a], Zhenzhen Xue[b], Hongli Chen[c], Hongwei Wu[a], Tianyun Wang[d]

[a]Key Laboratory of Medical Molecular Probes, School of Basic Medical Sciences, Xinxiang Medical University, Xinxiang, Henan 453003 China

[b]School of Pharmacy, Xinxiang Medical University, Jinsui Road 601, Xinxiang, Henan 453003, China

[c]School of Life Sciences and Technology, Xinxiang Medical University, Jinsui Road 601, Xinxiang, Henan 453003 China

[d]Department of biochemistry, Xinxiang Medical University, Jinsui Road 601, Xinxiang, Henan 453003, China

*Corresponding author: Tel +86-373-3029128, Fax +86-373-3029959

E-mail: xuefangshang@126.com

Table (S1). Selected Bond Lengths (Å) and Angles (°) for the Compound **2**

Selected bonds	Bonds length and bonds angles
O(3)-C(7)	1.355(2)
O(3)-C(8)	1.407(2)
C(7)-O(3)-C(8)	120.78(16)

Table (S2). Hydrogen bonds for Compound **2**

D-H...A	d(D-H) (Å)	d(H...A) (Å)	d(D...A) (Å)	<(DHA) (°)
C(3)—H(3) ..F(1)	0.93	2.33	2.6631	101
C(10)—H(10) ..O(5)	0.93	2.46	3.3666	166

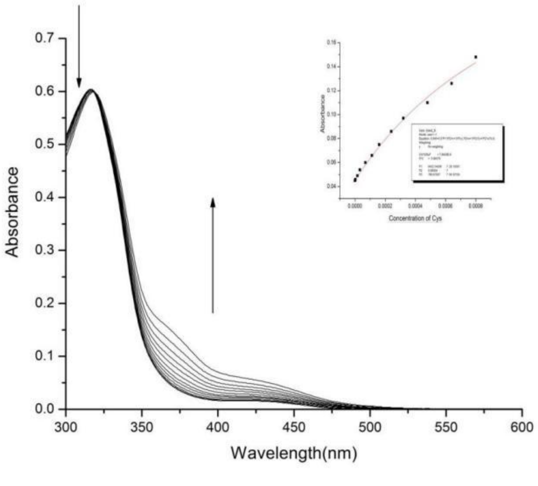

Figure (S3). UV-vis spectra of compound **1** (4.0×10^{-5} $mol\cdot L^{-1}$) with the addition of Cys ($0\text{-}8\times10^{-6}$ $mol\cdot L^{-1}$) in DMSO solution. Arrows indicate the direction of increasing Cys concentration.

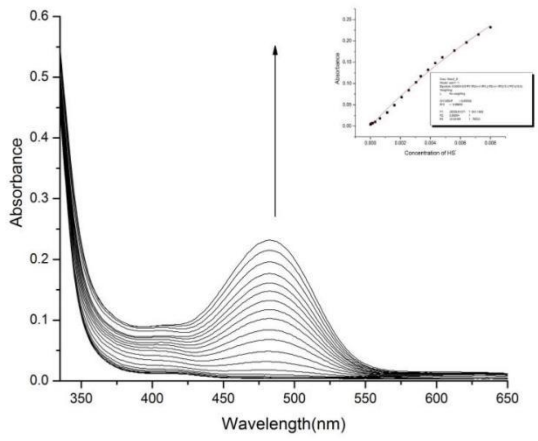

Figure (S4). UV-vis spectra of compound **2** (4.0×10^{-5} $mol\cdot L^{-1}$) with the addition of HS^- in DMSO solution. Arrows indicate the direction of increasing HS^- concentration.

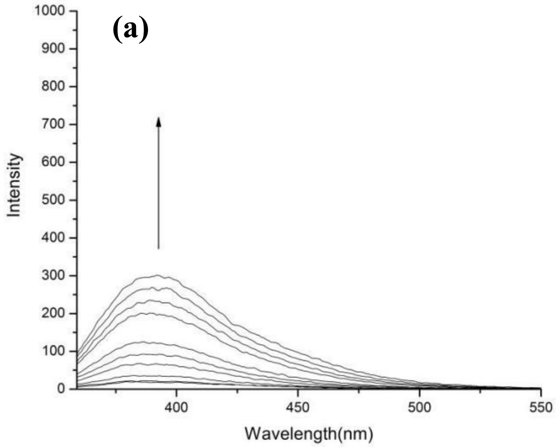

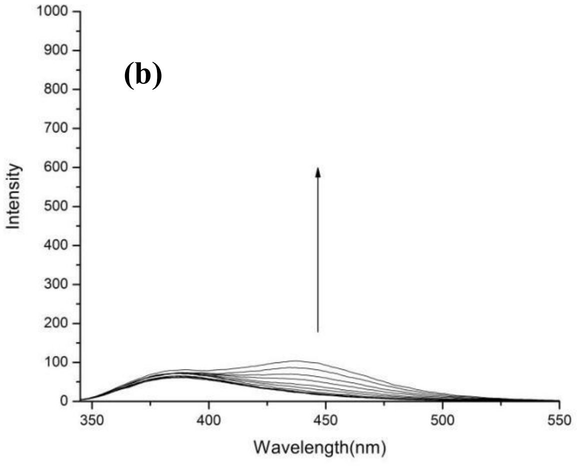

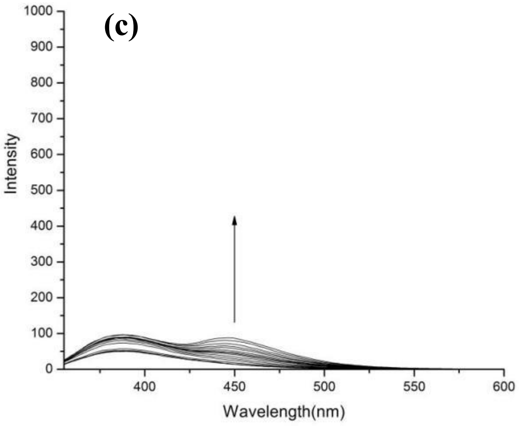

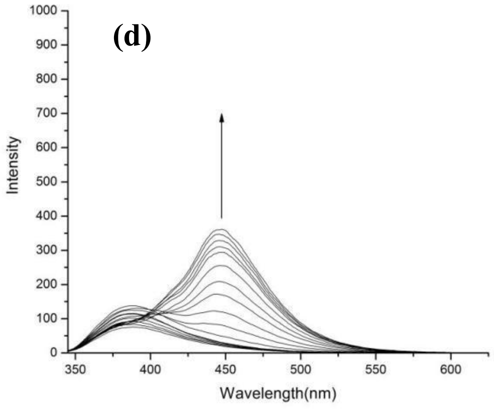

Figure (S5). Fluorescence responses (λ_{ex} 331 nm, slit widths: 5nm/5nm) of compound **1** (4.0×10^{-5} mol.L^{-1}) upon the additions of various anions and Cys ($(0\text{-}80)\times10^{-5}$ mol·L^{-1}) in pure DMSO solution. a) Cys, b) $H_2PO_4^-$,c) AcO^-, d) F^-. Arrows indicate the increasing concentration of anions and Cys.

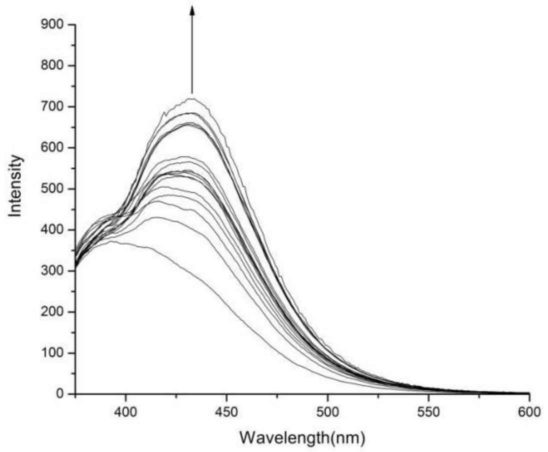

Figure (S6). Fluorescence response (λ_{ex} 348 nm, slit widths:10nm/10nm) of compound **2** (4.0×10^{-5} mol.L^{-1}) upon the addition of HS^- ($(0\text{-}80)\times10^{-5}$ mol·L^{-1}) in pure DMSO solution. Arrows indicate the direction of increasing HS^- concentration.

Synthesis of 7-hydroxy-4-methylcoumarin

Resorcinol (11g, 0.1 mol) was dissolved in concentrated sulfuric acid (160 mL) and then ethyl acetoacetate (13 mL, 0.1 mol) was added slowly under ice-water bath. The above mixture was reacted for 1 h placed ice-water bath, and then for 24 h at room temperature. The reaction was poured into ice bath with vigorous stirring, a yellow crude was obtained after filtration. Then the precipitate was recrystallized from ethanol and dried under vacuum. Yield: 85 %. m.p. 187.5-188.5°C. ^{1}H NMR (400 MHz, DMSO-d_6) δ 10.53 (s, 1H, -OH), 7.59 (t, 1H, 5-H), 6.80 (s, 1H, 8-H), 6.71 (s, 1H, 6-H), 6.14 (s, 1H, 3-H), 2.37 (s, 3H, $-CH_3$) **(supplementary material)**; MS-HRMS (m/z): 199.0368 $(M+Na)^+$ **(supplementary material)**.

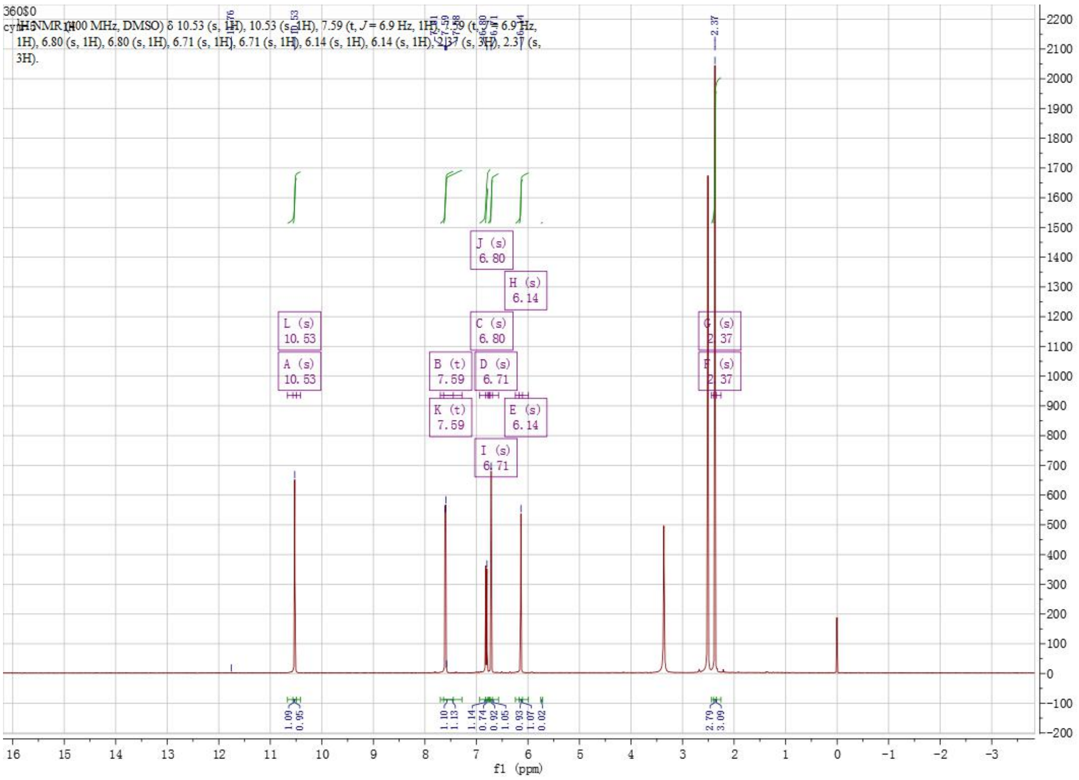

Figure (S7). ^{1}H NMR spectrum of 7-hydroxy-4-methylcoumarin.

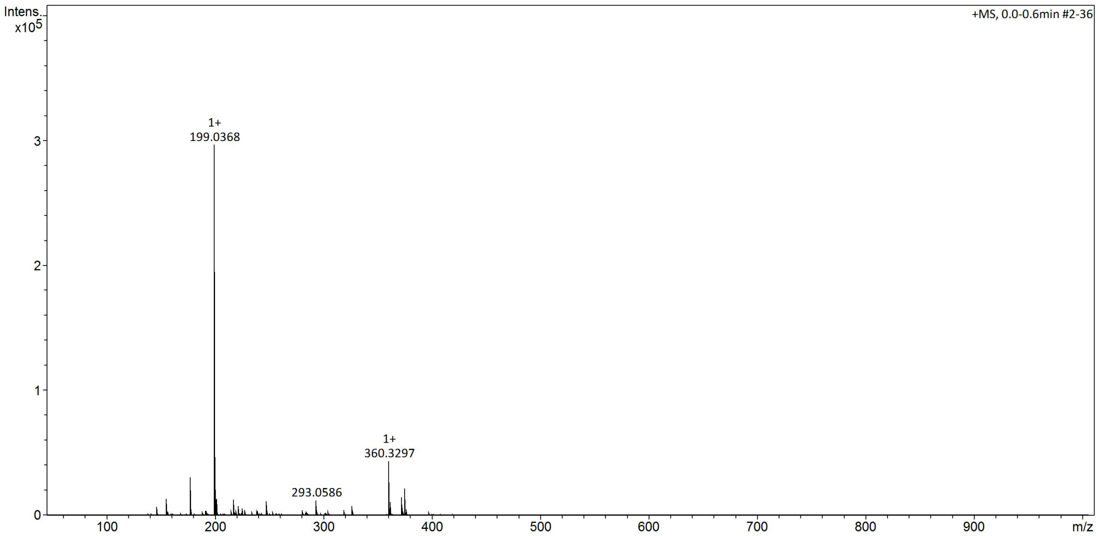

Figure (S8). MS-HRMS spectrum of 7-hydroxy-4-methylcoumarin MS-HRMS (m/z):199.0368(M+Na)$^+$

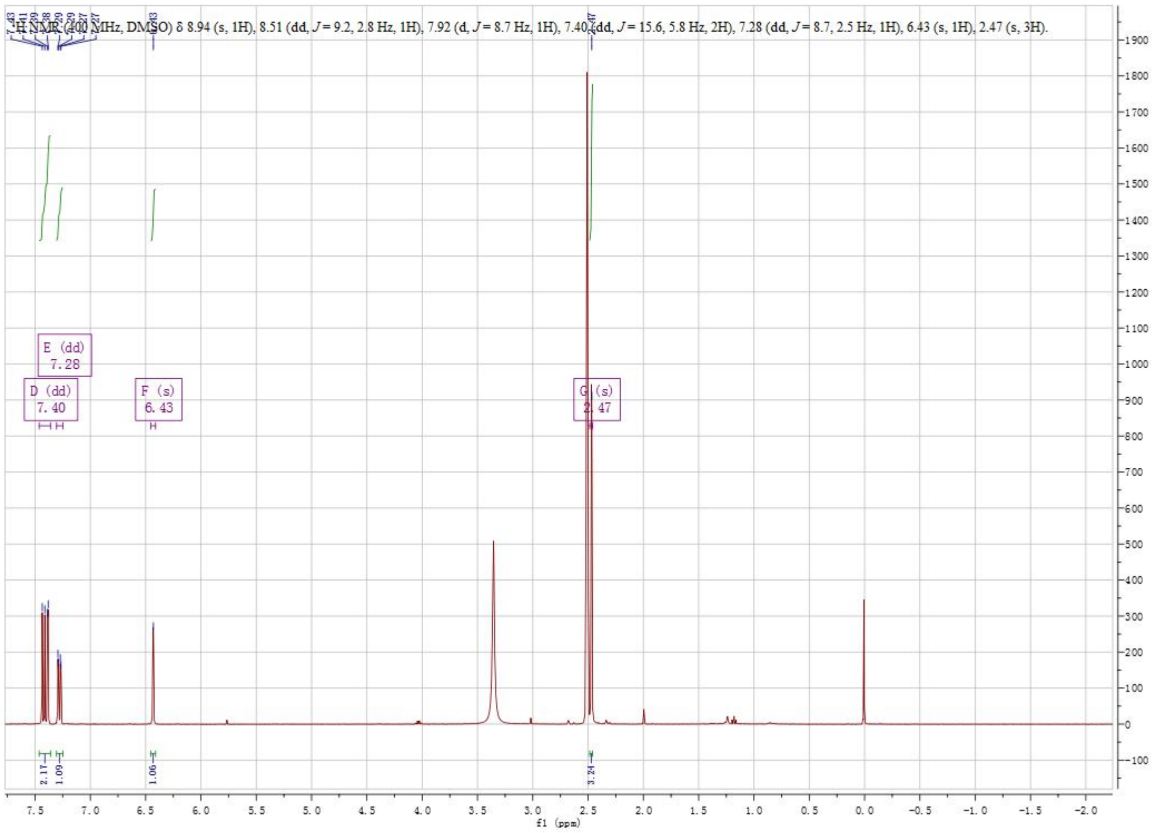

Figure (S9). ^{1}H NMR spectrum of compound **1**.

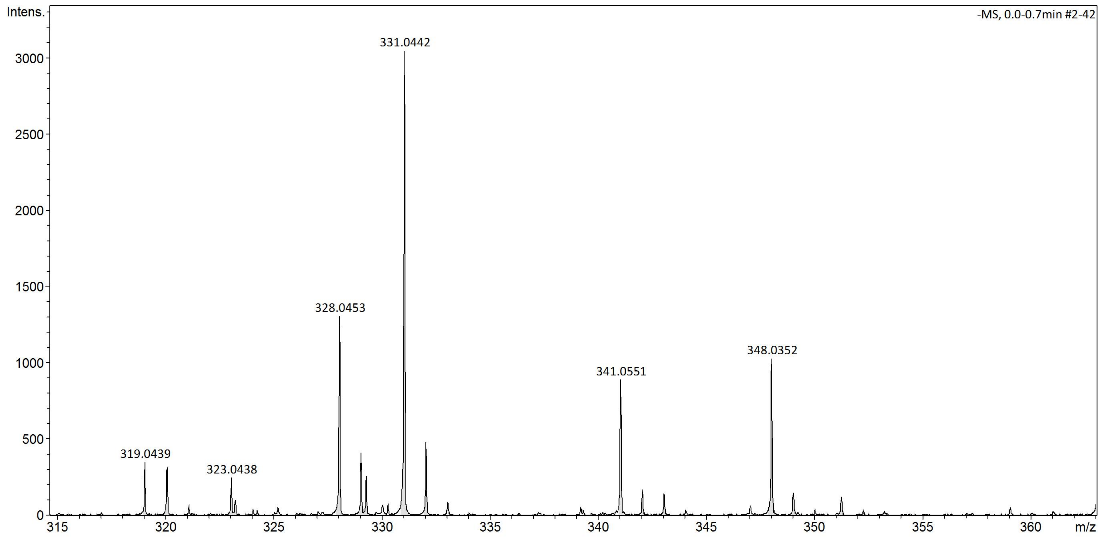

Figure (S10). MS-HRMS spectrum of compound **1** MS-HRMS (m/z):341.0551(*M*-H)$^{-}$

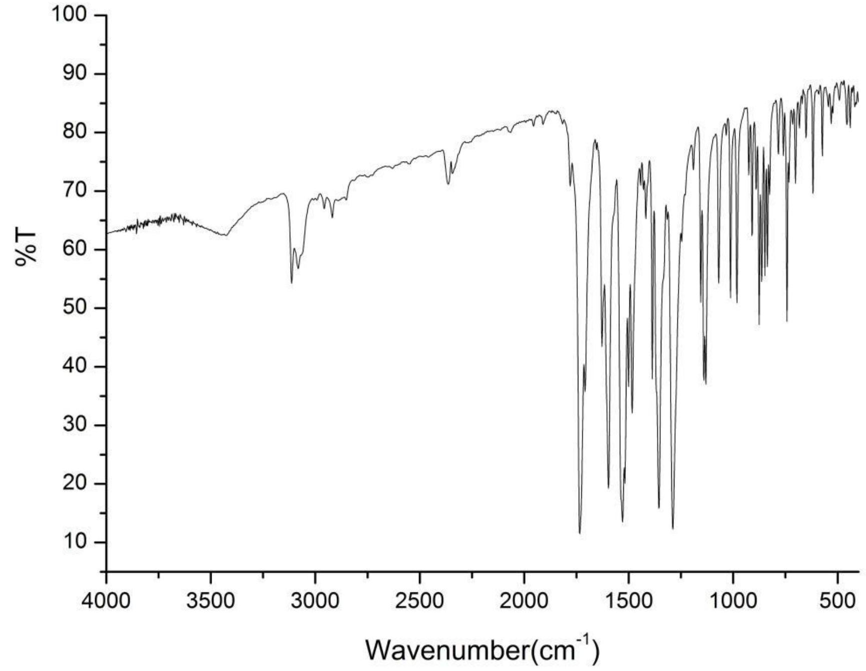

Figure (S11). IR spectrum of compound **1** in KBr pellet.

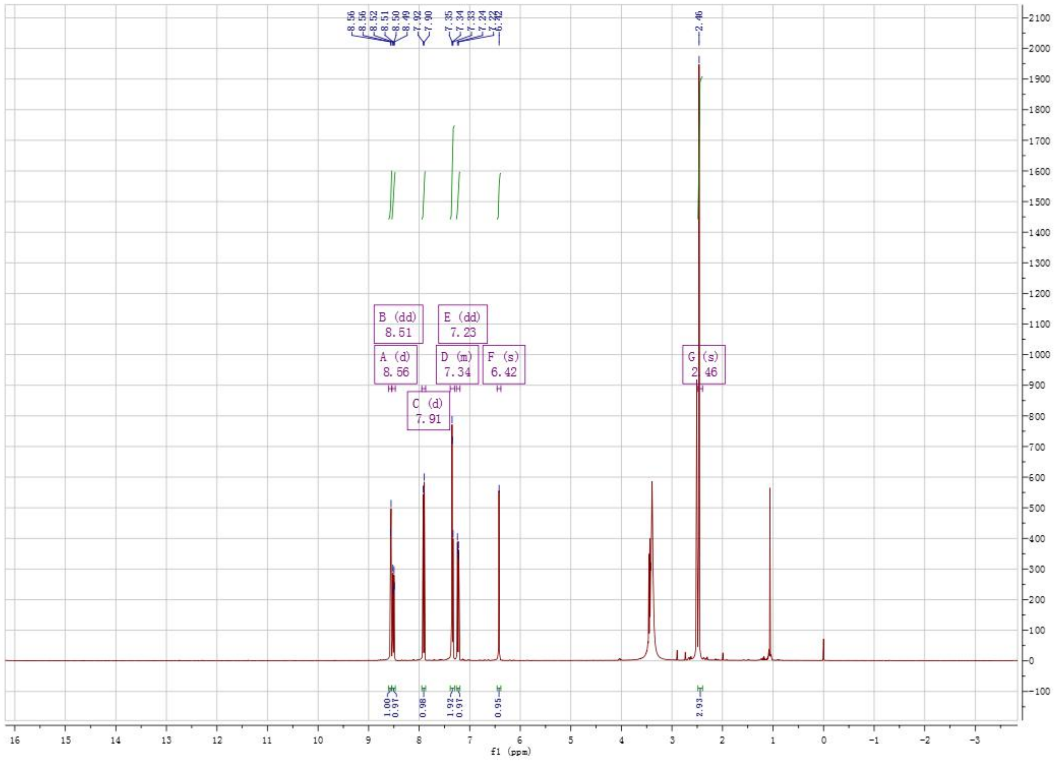

Figure (S12). ^{1}H NMR spectrum of compound **2**.

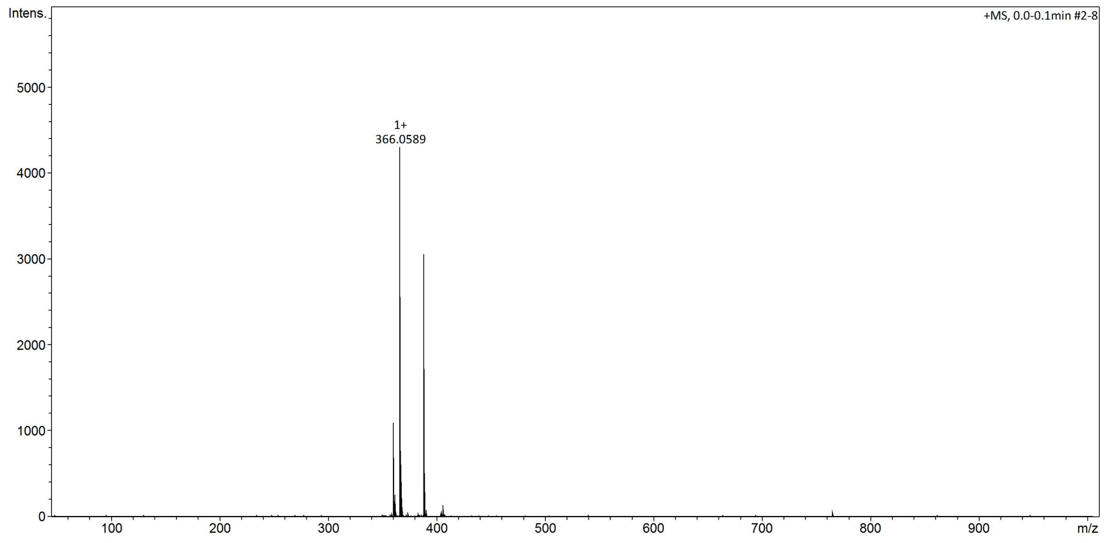

Figure (S13). MS-HRMS spectrum of compound **2** MS-HRMS (m/z): 366.0589(M+H)$^+$

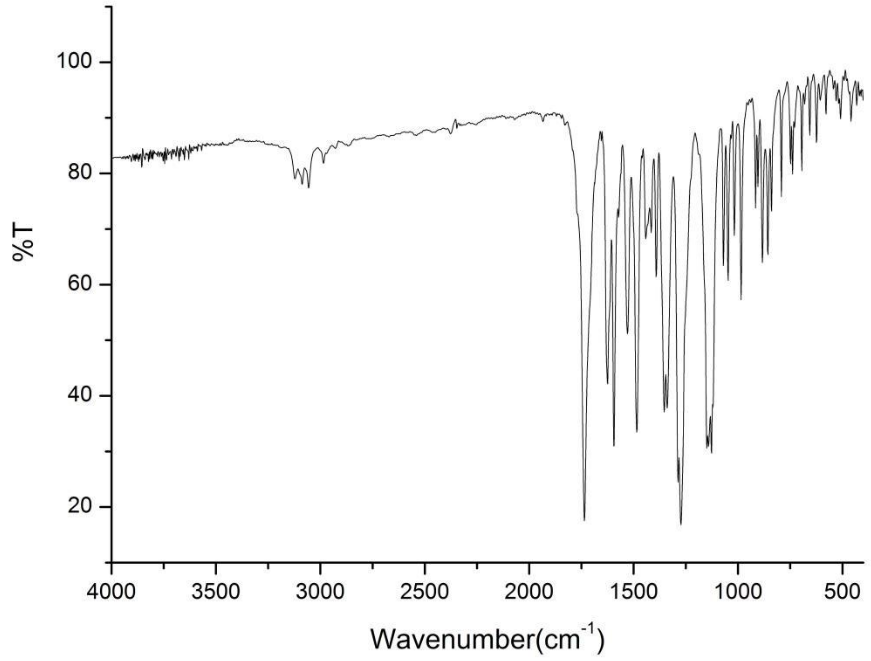

Figure (S14). IR spectrum of compound **2** in KBr pellet.

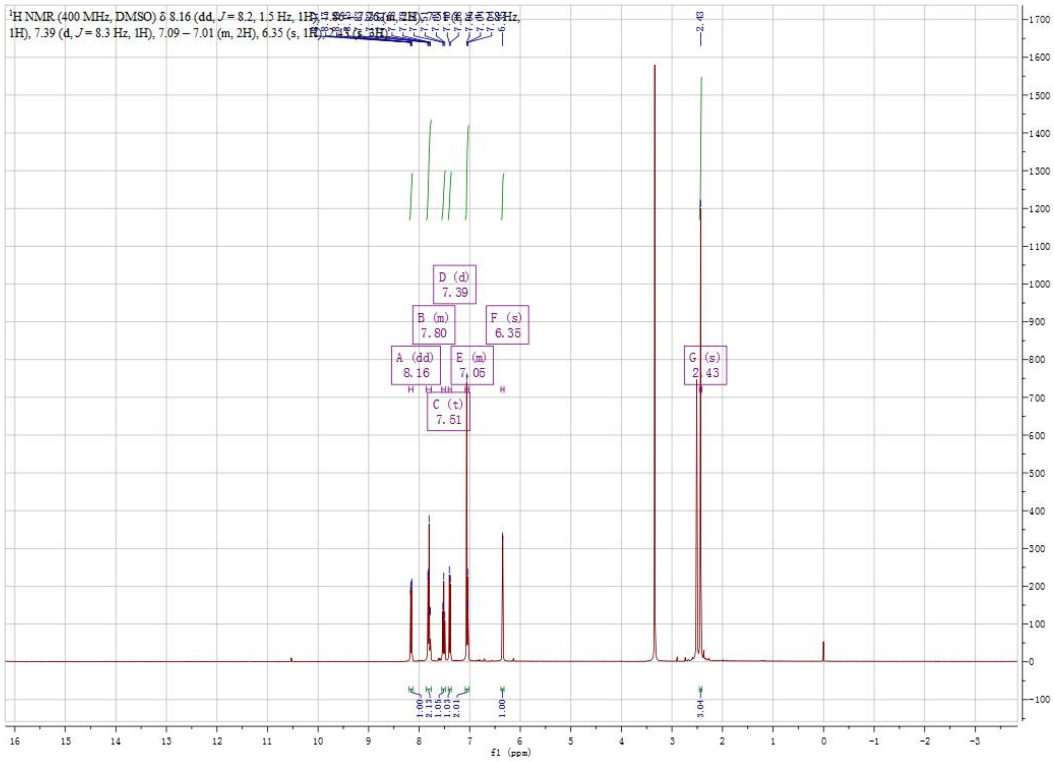

Figure (S15). ^{1}H NMR spectrum of compound **3**.

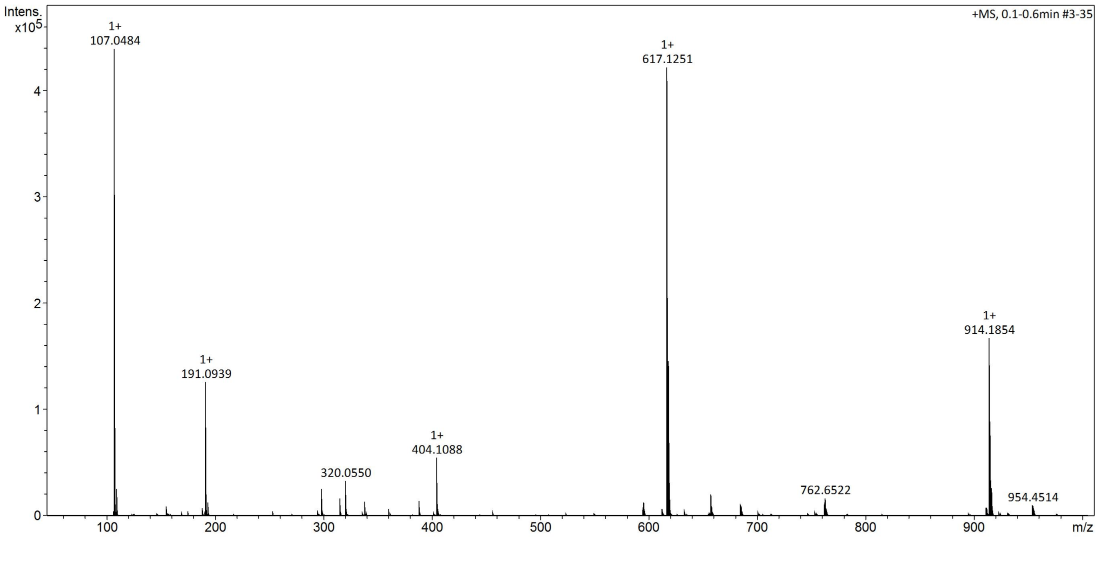

Figure (S16). MS-HRMS spectrum of compound **3** MS-HRMS (m/z): 320.0550(M+Na)$^+$

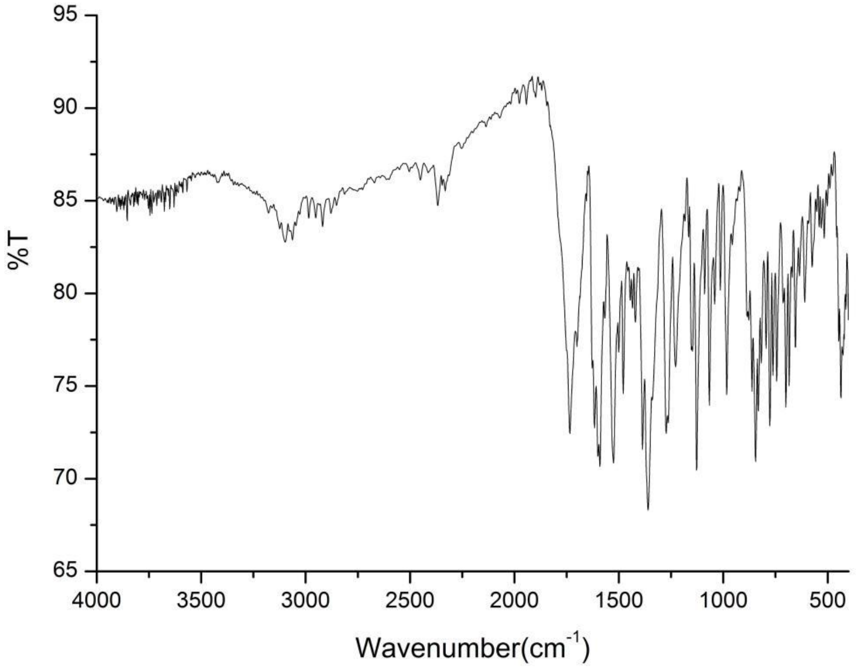

Figure (S17). IR spectrum of compound **3** in KBr pellet.

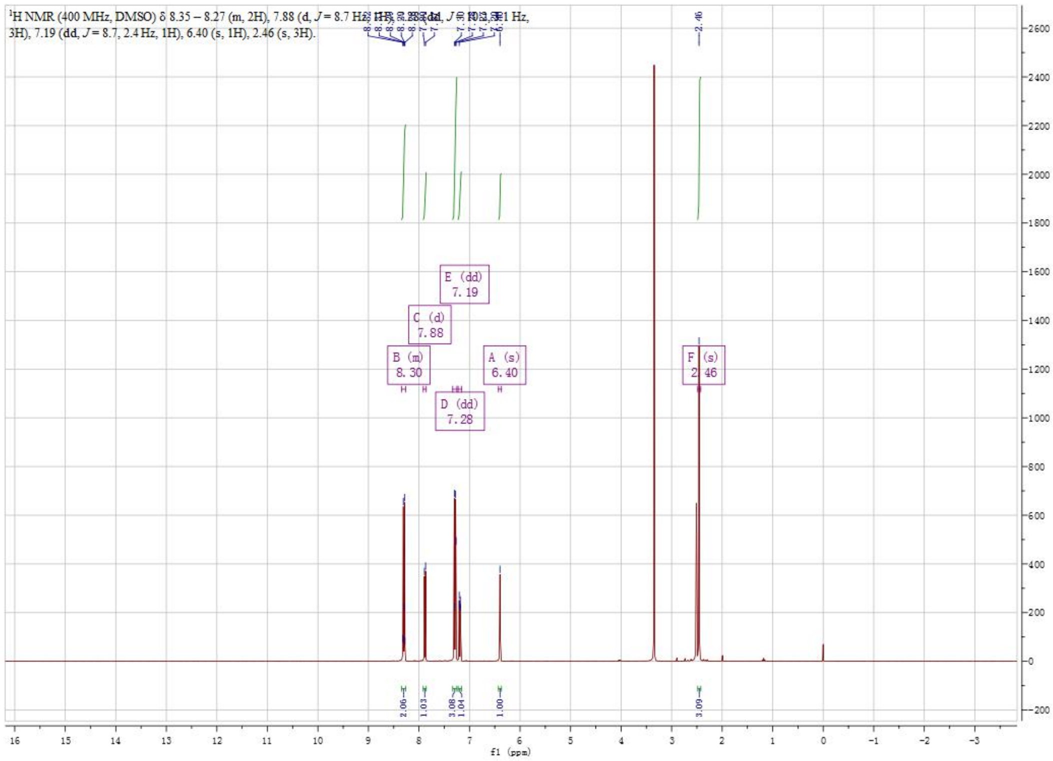

Figure (S18). ^{1}H NMR spectrum of compound **4**.

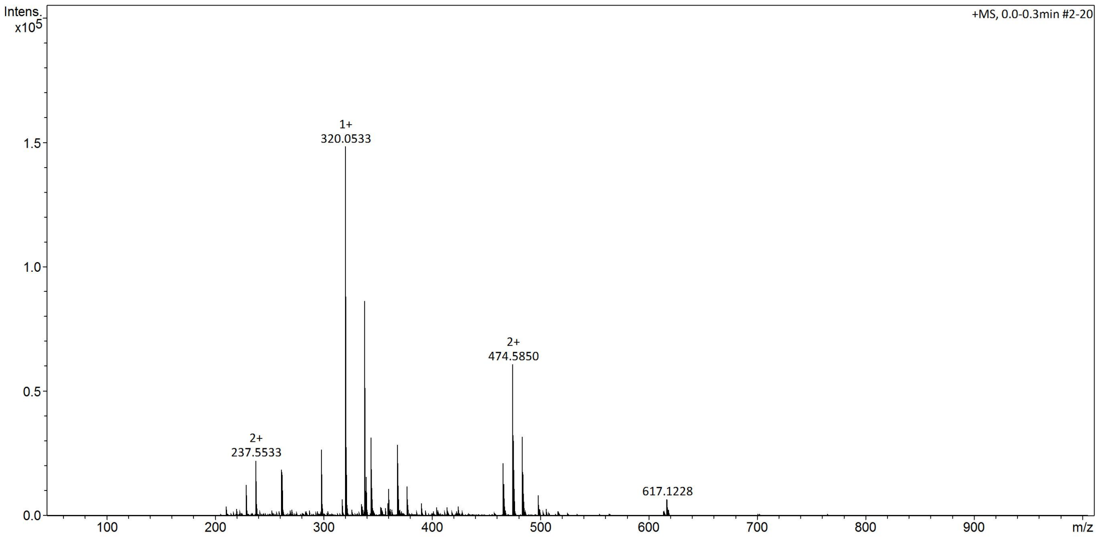

Figure (S19). MS-HRMS spectrum of compound **4** MS-HRMS (m/z) :320.0533 (M+Na)$^+$

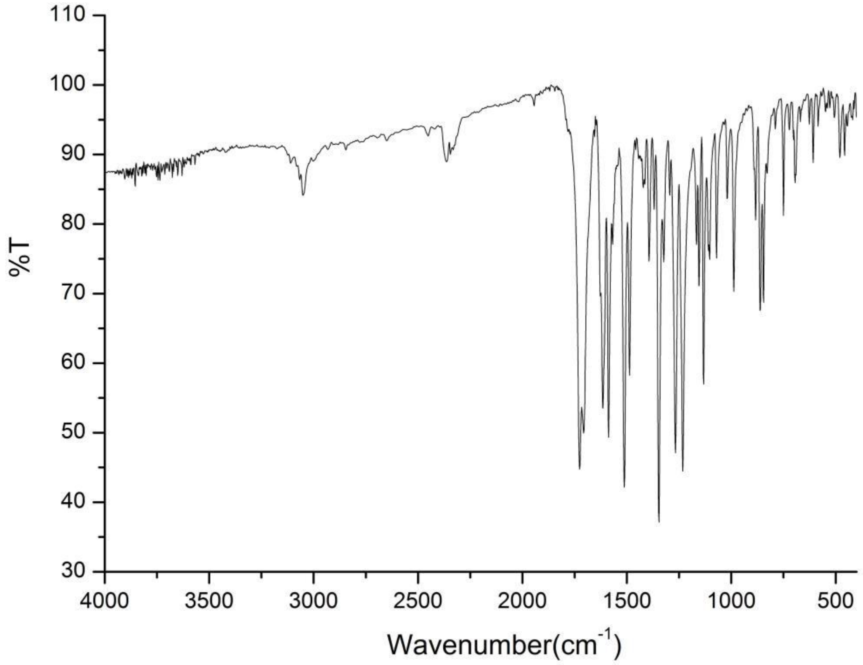

Figure (S20). IR spectrum of compound **4** in KBr pellet.
